# Cost-Effectiveness of Pertussis Vaccination Schedule in Israel

**DOI:** 10.3390/vaccines9060590

**Published:** 2021-06-02

**Authors:** Dean Langsam, Dor Kahana, Erez Shmueli, Dan Yamin

**Affiliations:** Department of Industrial Engineering, Tel-Aviv University, Tel-Aviv 6997801, Israel; deanla@gmail.com (D.L.); kahana.d@gmail.com (D.K.); erez.shmueli@gmail.com (E.S.)

**Keywords:** transmission model, vaccine allocation, pertussis, waning immunity, population model, cost-effectiveness analysis

## Abstract

Pertussis is a highly contagious bacterial disease that primarily affects infants. To optimize the pertussis vaccination schedule in Israel and evaluate the cost-effectiveness of alternative strategies that add or remove booster doses, we developed an age-structured model for pertussis transmission. Our model was calibrated using 16 years of data from laboratory-confirmed pertussis cases in Israel. Costs and quality-adjusted life years (QALYs) projected by the model within 12 years from the implementation of the considered interventions were compared with the current vaccination schedule. We found that by using the same number of vaccines administered today, the targeting of children at the age of six instead of seven would be predicted to be the optimal schedule to decrease both outpatient visits and hospitalizations. We also found that any increase in maternal vaccination coverage is likely to be cost-effective, with an incremental cost-effectiveness ratio of $77,000–$97,000 per QALY. By contrast, the contribution of the second booster dose is limited, with a probability of only 0.6 to be cost-effective at $110,000/QALY saved. Additional effort should be invested to encourage maternal vaccination against pertussis. We recommend moving the first booster to age six and prudently considering the necessity of the second booster dose.

## 1. Introduction

Over the past two decades, a resurgence of pertussis has been observed in several countries [1,2,3,4]. Pertussis, also known as whooping cough, is a highly infectious disease of the upper respiratory tract that is caused by the Gram-negative bacillus *Bordetella pertussis*. Pertussis is so contagious that, prior to the vaccination era, nearly all individuals became infected at least once throughout their lifetime [5]. Children under the age of five are most vulnerable to pertussis, and the disease accounts for approximately 160,000 deaths worldwide annually, most of which occur in developing countries [6]. In developed countries, pertussis infection is associated with a substantially lower mortality rate, but morbidity remains high. In the United States alone, there are roughly 21,000 reported pertussis cases per year and approximately 2000 hospitalizations due to pertussis annually [7].

The recent increase in the incidence of pertussis is widely believed to be a consequence of the change from the whole-cell vaccine (WCV) to the acellular vaccine (ACV) [8]. The antibody levels induced by WCV are efficiently boosted compared to ACV [9,10], and memory B cells persist longer [11]. Furthermore, pertussis-specific T-cell responses persist longer after WCV compared to ACV [12].

Several studies have suggested that this change may result in a more rapid waning of the immunity that could be as short as four years [13,14,15,16]. Other studies have argued that the ACV variant efficiently protects against the symptoms of pertussis but is less effective at preventing pertussis transmission [17,18,19].

The policy for immunization against pertussis in Israel was primarily based on the general guidelines adopted worldwide. Due to the increase in cases and particularly in hospitalizations, Israel added two booster doses: one given to 13-year-old children, added in 2006, and a second for 7-year-old children, added in 2008. However, despite the high overall vaccination coverage against pertussis, the incidence of pertussis after this time rose steadily. Recently, the maternal Tdap vaccination was shown to substantially reduce the risk of pertussis infection for both the mother and her infant [20]. The maternal Tdap vaccine stimulates the development of maternal pertussis antibodies, which are passed through the placenta and protect the newborn against pertussis in early life. In addition, the vaccine protects the mother around the time of delivery, making her less likely to become infected and transmit pertussis to her infant [21]. Consequently, nationwide recommendations that pregnant women become vaccinated emerged in several developed countries, including in the United Kingdom [20] and in the United States [22]. In Israel, the maternal Tdap vaccine was recommended and funded by the Ministry of Health in January 2015 [23]. Recently, a retrospective study in Israel suggested that a sharp decline in hospitalizations of young unvaccinated infants was likely to be attributed to vaccination during pregnancy [24]. However, the effect of this policy on the population level remains unclear, particularly due to the multi-year periodicity of pertussis demonstrated in 64 countries [25] and in Israel [24] and due to the changing efficacy of the vaccine due to waning immunity.

Mathematical models for disease transmission are widely used to evaluate the effectiveness of vaccination programs [26,27]. Several transmission models have been proposed for pertussis [3,13,16,19,28,29]. These models identified the drivers and the extent of periodicity [24,30], explored the impact of asymptomatic infections on transmission [19], and quantified the waning period of WCV and ACV [13,16]. One recent study demonstrated that including one dose of WCV instead of ACV can be highly economical [19]. Recently, case-control studies have shown that a maternal booster for pregnant women dramatically reduces disease in newborns by providing a stronger maternal immunity [20] or by the cocooning effect; i.e., by reducing mild disease in parents [26]. However, no model to date has evaluated the population-level effectiveness of maternal pertussis vaccination in Israel by accounting for herd protection. In addition, to the best of our knowledge, no model has sought to optimize the pertussis vaccination schedule following the recent rise in incidence.

In this work, we developed an age-stratified compartmental transmission model fitted to primary data of laboratory-confirmed cases of pertussis in Israel. We then used this model to evaluate the effectiveness and cost-effectiveness of the two recent additions to the vaccination policy: (1) age-specific booster doses and (2) maternal vaccination.

## 2. Materials and Methods

### 2.1. Transmission Model

**Figure 1:**
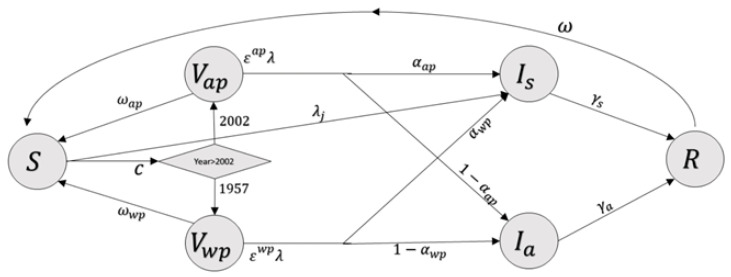
Compartmental diagram of the transmission model. Individuals are born in a susceptible compartment *S*, where they can become symptomatically infected *Is*. Following infection, individuals transition to a recovered compartment *R*, where they are temporarily immune. Following immunity waning, individuals return to *S*. Depending on the year and their age, individuals are vaccinated with a whole-cell vaccine, *Vwp*, or an acellular vaccine, *Vap*. Vaccinated individuals are at a reduced risk of becoming infected, and if infected can be either symptomatic, *Is*, or asymptomatic, *Ia*. For clarity, age stratification is not shown in the figure.

We developed a transmission model for age-stratified pertussis infection in Israel (Figure 1). The model uses a modified susceptible–vaccinated–infected–recovered (SVIR) framework [31], where individuals transition between categories over time according to their age and health status. The model accounts for the change in vaccine uptake in Israel from 2002 by considering different protections gained from the whole-cell vaccine versus the acellular vaccine. Because there was no vaccination for pregnant women until 2015, the individuals included in this study were born with no maternal immunity.

Specifically, we stratified the population into 27 age groups: 0–2 months, 2–4 months, 4–6 months, 6–8 months, 8–10 months, 10–12 months, 1–2 years, 2–3 years, 3–4 years, 4–5 years, 5–6 years, 6–7 years, 7–8 years, 8–9 years, 9–10 years, 10–11 years, 11–12 years, 12–13 years, 13–15 years, 15–18 years, 18–21 years, 21–25 years, 25–35 years, 35–45 years, 45–55 years, 55–65 years and >65 years. In line with previous studies [19,32], infected individuals can be either symptomatic or asymptomatic. Individuals in each age group in the model were divided into six health compartments: susceptible, symptomatic infectious, asymptomatic infectious, whole-cell vaccinated, acellular vaccinated, and recovered.

Chronologically, individuals are born susceptible to the disease. As individuals age, they either become vaccinated with one of the two types of vaccines (depending on the vaccination year) or remain susceptible. Both vaccinated and unvaccinated individuals can become infected with a reduced probability compared to unvaccinated individuals. However, vaccinated individuals will profit from a reduction in the infection probability, which depends on the efficacy of the vaccine they received. An infected individual may be either symptomatic or asymptomatic and will recover at some rate. Recovered individuals are immune to reinfection but lose their natural immunity at some rate, after which they return to being susceptible. The full model and equations of the transmission model are detailed in the Supplemental Information.

#### 2.1.1. Calibration of Unknown Parameters

The model includes both fixed, previously known parameters that were obtained from the literature (Table 1) and unknown free parameters (Table 2). To empirically estimate the unknown epidemiological parameters, we calibrated our model according to the monthly cases of pertussis, stratified by age into three “parent” age groups (SI): 0–1 years, 1–21 years and >21 years. The data for this calibration were provided by the Israeli Ministry of Health and include reported cases of pertussis over a period of 16 years (1998–2013). Reported cases were collected in either outpatient visits or hospitalizations and were laboratory-confirmed either by PCR or by serological tests. Because not all pertussis cases are reported, we estimated the total number of cases by calculating the number of reported cases divided by the reporting ratio (for a given age group). The reporting ratio for a given age group was extracted from a previous study, which used serological tests conducted in Israel to compare the number of reported cases to the total number of cases [33].

To properly assess the distribution of the periodical offset ϕ and the three susceptibility rates $f_{j}$ (corresponding with the “parent” age groups), we deployed a Markov Chain Monte Carlo algorithm. The constant susceptibility ρ was selected by a scenario analysis to best describe a maximum (log-) likelihood and the convergence of other parameters; meanwhile, the approach was robust to changes both in terms of the convergence of the free parameters and the prediction simulation.

#### 2.1.2. Model Projections

We first drew 2000 samples of the parameters of the four models from the joint probability distribution (SI). For each sampled set of parameters and each vaccination policy, we then simulated the model and projected pertussis infections for each age group over a 12-year time horizon. In each simulation of the dynamic model, we recorded the daily number of vaccinated and infected individuals that were stratified by age group.

### 2.2. Clinical Outcomes

We used the projection of the daily number of infected individuals from our dynamic model to compute four clinical outcomes: (1) home treatment, (2) outpatient visits, (3) hospitalization, and (4) death. The probability that a case of pertussis for a given age group would lead to hospitalization was estimated using data from reported cases of pertussis in Israel [24]. Death is a rare event. To account for the age-related probability of death after infection, we adopted a similar assumption of a previous US study [29]. These outcomes were integrated into an economic evaluation to estimate the effectiveness and cost-effectiveness of different pertussis vaccination policies.

### 2.3. Quality of Life and Costs

Costs were compiled from available published sources in Israel (Table 2). Quality-adjusted life-years losses (QALYs) were evaluated by multiplying the daily discomfort levels of infection compared to no infection with the number of discomfort days. Thus, as was consistent with previous CEA studies [29], for an infection resulting in home treatment, we considered 0.0022 QALYs, corresponding to a disutility of 0.1 per day for an 8-day period. For an outpatient visit, we considered 0.0022 QALYs, corresponding to a disutility of 0.1 per 25 day period. For hospitalizations, we considered an additional disutility of 0.42 days for a 4 day period, which is the mean hospitalization period of pertussis episodes in Israel [24]. Because pertussis-related mortality typically occurs only in young infants, we assumed a loss of 80 years, which corresponds to the mean life expectancy in Israel (Table 3). For comparison with other vaccination policies in Israel, we present the incremental cost-effective ratio. In our base case, all costs and QALYs were discounted at a rate of 3.5%, which is in accordance with previous studies [35,36,37]. The source code of the model, calibration, and simulation studies are available online [38].

### 2.4. Policy Optimization and Cost-Effectiveness Analysis

The cost-effectiveness analysis of medical intervention presents the balance between the cost of the intervention and the incremental health benefits attributable to the intervention. We calculated the cost per QALY saved by vaccination to quantify the cost of purchasing a year of good health. We used the terminology suggested by the WHO that defines “cost-effective” as lower than three times the annual per capita gross domestic product (GDP) and “very cost-effective” as lower than the GDP.

We used our calibrated model to evaluate the effectiveness and cost-effectiveness of several different potential vaccination approaches. First, we optimized the vaccination age of the two existing booster doses (currently provided at ages 7 and 13) concerning decreasing outpatient visits and hospitalizations. We then extended our analysis to evaluate the effect of (1) removing a booster dose, (2) adding a booster dose, and (3) increasing vaccination coverage among pregnant women. In each of these analyses, we first identified the optimal vaccination schedule for reducing pertussis outpatient visits and hospitalizations and then determined the incremental cost-effective ratio of the optimal schedule from the societal perspective.

### 2.5. Sensitivity Analysis

To confirm the robustness of our findings, we accounted for uncertainties of the reported incidence of pertussis by sampling 2000 samples of four model parameters from the joint probability distribution. We also used data on hospitalized patients between 2004–2014 to account for the uncertainty of hospitalization given infection in each sample (SI). As the actual vaccination coverage among pregnant women is unknown, we determined the cost-effectiveness of such a policy considering coverage of 25%, 50%, 75%, and 100%. Furthermore, we evaluated the probability of a policy being cost-effective, assuming a willingness to pay ranging between 0 and USD 200,000 per QALY gained. The code for the model, the calibration using Markov Chain Monte Carlo, the simulations, and the statistical analyses were written in Python 3.7.

## 3. Results

### 3.1. Model of Pertussis Transmission

**Figure 2:**
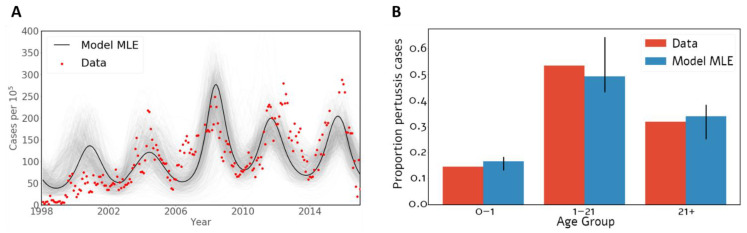
Fit of the model. (**A**) Time series of recorded monthly pertussis cases and model fit, with the solid line representing the maximum likelihood. (**B**) Data and model fit to the age distribution among pertussis infections.

Our pertussis transmission model was calibrated to the age-stratified monthly incidence of pertussis in Israel (Figure 2). With only four free parameters, the model recapitulated the pertussis monthly trends (Figure 2A) and age distribution (Figure 2B) of symptomatically infected individuals. For example, the model captured the clear pattern of 4-year periodicity and the age distribution of pertussis cases.

### 3.2. Optimal Vaccination Schedule

**Figure 3:**
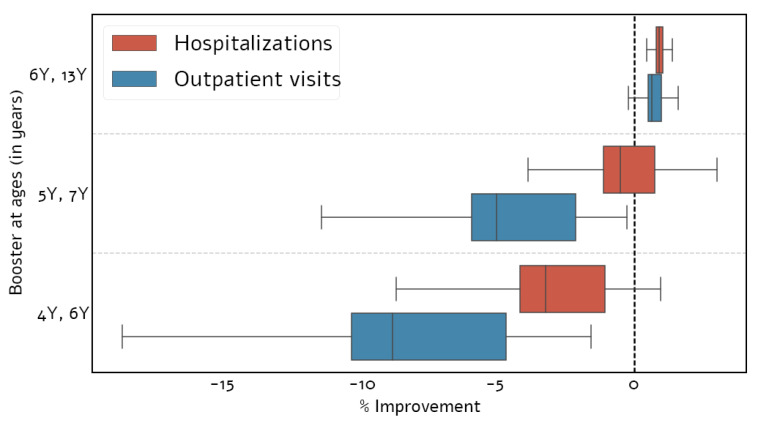
Effectiveness of different vaccination schedules for two booster doses. Effectiveness of different schedules in terms of reducing pertussis outpatient visits and hospitalizations for two booster doses as projected by our model. Effectiveness is reported as the percentage of improvement compared to the current vaccination schedule that targets children at the ages of 7 and 13.

Current pertussis vaccination policy in Israel calls for booster doses at ages 7 and 13. Using our model, we first optimized the timing of these booster doses. We found that shifting the age of the first booster dose from 7 to 6 yielded optimal outcomes, with respect to both outpatient visits and hospitalizations (Figure 3). Specifically, compared to the current policy, this strategy resulted in 0.64% (IQR: 0.31–1.12%) fewer outpatient visits and 0.92% (IQR: 0.70–1.22%) fewer hospitalizations, on average, per year.

### 3.3. Cost-Effectiveness Analysis

We then extended our analysis to evaluate the effects of (1) removing a booster dose, (2) adding a booster dose, and (3) increasing vaccination coverage among pregnant women. We first identified the optimal vaccination schedule for reducing pertussis outpatient visits and hospitalizations in each of these analyses. We then determined the incremental cost-effectiveness ratio of the optimal schedule.

#### 3.3.1. Existing Booster Doses

**Figure 4:**
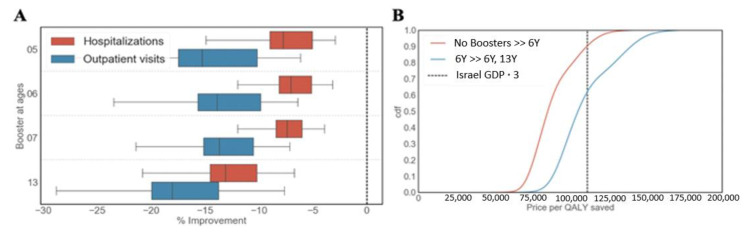
Cost-effectiveness of existing booster doses. (**A**) Effectiveness of different vaccination schedules of a single booster dose as projected by our model in terms of reducing pertussis outpatient visits and hospitalizations. Effectiveness is reported as the percentage of improvement compared to the optimal two-booster vaccination schedule that targets children at the ages of 6 and 13. (**B**) Cost-effectiveness of the optimal single-booster schedule compared to the no-booster schedule and the optimal two-booster schedule. The dashed vertical line represents $110,000, which corresponds to three times the per capita GDP of Israel.

We first identified the optimal vaccination schedule when allowing a single booster dose only. We found that targeting children at the age of six resulted in the best performance in terms of reducing the number of both outpatient visits and hospitalizations (Figure 4A).

We then conducted a cost-effectiveness analysis to compare the optimal single booster dose schedule to a no-boosters schedule. Our results suggest that, compared to the no-booster schedule, the optimal single-booster dose schedule (given at the age of 6) is cost-effective at $110,000/QALY with a probability of roughly 0.9 (Figure 4B). In contrast, we found that, compared to the optimal single booster dose schedule, the contribution of the optimal two-booster schedule (with boosters administered at the ages of 6 and 13) is low. Specifically, the two-booster schedule that targets children at the age of 6 and 13 is cost-effective at $110,000/QALY with a probability of 0.6 (Figure 4B).

#### 3.3.2. Adding a Booster Dose

**Figure 5:**
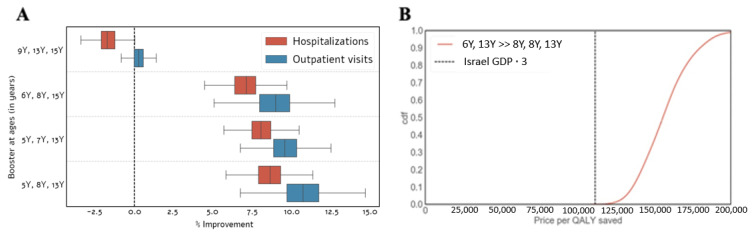
Cost-effectiveness of adding a third booster dose. (**A**) Effectiveness of different vaccination schedules with three booster doses in terms of reducing pertussis outpatient visits and hospitalizations as projected by our model. Effectiveness is reported as the percentage of improvement compared to the optimal two-booster schedule, which targets children at the ages of 6 and 13. (**B**) Cost-effectiveness of the optimal three-booster schedule compared to the optimal two-booster schedule.

We also analyzed the extent of improvement obtained with a three-booster schedule compared to a two-booster schedule at the ages of 6 and 13 (identified above as the optimal two-dose vaccination schedule). A schedule of administering booster doses at ages 5, 8, and 13 provided optimal reductions in the number of outpatient visits and hospitalizations (Figure 5A). It is worth noting that not all age combinations of three booster doses led to an improvement (Figure 5A). Our cost-effectiveness analysis suggests that the optimal policy (i.e., administering booster doses to children at the ages of 5, 8, and 13) is not cost-effective at $110,000/QALY with a probability of nearly 1.0 (Figure 5B).

#### 3.3.3. Maternal Vaccination

**Figure 6:**
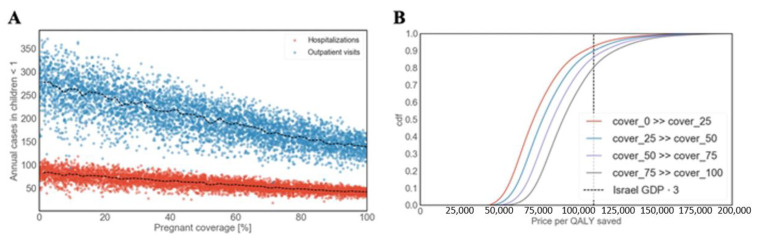
Cost-effectiveness of maternal vaccination. (**A**) Projection of pertussis outpatient visits and hospitalizations for different vaccination uptake in pregnant women based on our model. Results are shown for children in their first year of life. (**B**) Cost-effectiveness of increasing maternal vaccination coverage starting at four different coverage levels.

The recommendation to vaccinate pregnant women against pertussis is a relatively new policy, and as such, the actual adherence is unclear. Thus, we first analyzed the potential gain of the maternal vaccine by exploring vaccination uptakes ranging from 0–100%. We found that maternal vaccination can dramatically reduce both outpatient visits and hospitalizations in children during their first year of life (Figure 6A). More specifically, with 100% coverage of pregnant women, 138 outpatient visits and 41 hospitalizations can be averted annually in children over the age of 1 (Figure 6A). These results correspond to a 50% reduction, supporting the importance of the new recommendation. Furthermore, as projected by our model, even a relatively small increase in vaccination coverage can significantly reduce morbidity. For example, if we assume that 50% of pregnant women are currently being vaccinated, promoting the policy to achieve a total of 75% uptake will result in 37 fewer outpatient visits (18%) and 11 fewer hospitalizations (19%) in children over the age of 1. This change corresponds to a reduction of 60 outpatient visits (3%) and 11 hospitalizations (12%) in the entire population. Our cost-effectiveness analysis suggests that targeting pregnant women is cost-effective at $110,000/QALY with a probability that ranges between 0.8–0.95, depending on the vaccination coverage (Figure 6B). For example, increasing the vaccination coverage from 0% to 25% is cost-effective at $110,000/QALY with a probability of 0.95, while increasing the coverage from 75% to 100% is cost-effective with a probability of 0.8. This result indicates that, while the marginal contribution of additional vaccinations is decreasing, it is still cost-effective with a very high probability. Our results were found to be robust over a wide range of realistic settings (see Supplemental Information).

## 4. Discussion

The recent increase in the incidence of pertussis requires a more comprehensive assessment of the current vaccination policy. We developed a mathematical model for disease transmission to optimize pertussis vaccination schedules in Israel. Our main finding is that maternal vaccination is the most cost-effective strategy tested regardless of the vaccination coverage. Because over 88% of hospitalizations due to pertussis infection occur during the first year of life, maternal vaccination is pivotal in reducing hospitalizations and improving economic outcomes. This result is consistent with previous studies [20,28,41] and emphasizes the importance of promoting vaccination during pregnancy.

We also found that assuming the same number of vaccinations to be administered as today, targeting children at the age of six instead of seven for a booster is projected to be optimal in terms of reducing the number of outpatient visits and hospitalizations. Interestingly, this earlier booster is also more effective in reducing outpatient visits and hospitalizations for children under the age of six, as it reduces pertussis transmission. This finding can likely be attributed to the typical family demographics in Israel, where the gap between siblings does not exceed 7 years. Thus, a booster dose for six-year-old children results in slightly better outcomes. Nevertheless, moving the booster dose even earlier (e.g., to 5-year-old children) was not projected by our model to yield a better outcome, because 5-year-olds remain sufficiently protected four years after vaccination.

We also found that a second and third booster vaccine can further reduce both hospitalizations and outpatient visits. However, our model predicts that the costs of these interventions may outweigh their benefit. It may seem counterintuitive to eliminate a booster dose in light of the recent rise in the incidence of pertussis. Nevertheless, our transmission model for all realistic vaccination strategies indicated that asymptomatic and mild infections would remain substantial in all age groups. Accordingly, our economic model reveals that the optimal strategy would be to indirectly or directly target young children, as they are at an elevated risk for more severe outcomes once infected. Such prioritization is possible by vaccinating the mother during pregnancy or by vaccinating risky contacts.

This study has several limitations. First, our model assumes that the transmissibility of asymptomatic infections is the same as that of symptomatic cases. However, asymptomatic and symptomatic infections differ greatly; asymptomatic infection is usually associated with a lower bacterial load, but it involves more exposure as the individual is usually more active.

Second, the calibration of our model is based on reported rather than actual data using seroprevalence adjustments [33]. Moreover, a significant part of this data was collected before Israel replaced the WCV with the ACV. In order to address this issue, we conducted an extensive sensitivity analysis that accounted for the relatively high variance in the transmission model, and our findings remained consistent for all realistic settings.

Third, our model, and therefore our insights, are calibrated to data about Israel. However, Israel substantially differs from other developed countries regarding its environment, demographics, and vaccination adherence. All these factors have a substantial effect on the transmission dynamics. For example, the extremely high fertility rate in Israel may be one of the main reasons for the observed impact of maternal vaccination. Therefore, revisiting existing vaccination policies on a country-specific basis is essential.

## 5. Conclusions

Vaccinating pregnant women against pertussis was found to be the best strategy to reduce outpatient visits and hospitalizations. Because this is already Israel’s policy, additional efforts should be invested to encourage pregnant women to vaccinate against pertussis. By contrast, we recommend prudently considering the second booster dose, as its cost may outweigh its benefit. Moreover, administering a booster dose to children at the age of 6 instead of the current age of 7 seems to be the optimal schedule for reducing outpatient visits and hospitalizations. This schedule improves outcomes for both children in those corresponding age groups and in the broader population, due to reduced transmission. Changing the current policy to be consistent with this optimal schedule could be done with relatively little effort and cost and therefore should be positively considered.

## Figures and Tables

**Table 1:** Fixed parameters.

Parameter	Description	Value	Justification
Δ	Birth rate	Time dependent, about 2% yearly	[34]
$\mu_{j}$	Death rate	Time dependent	[34]
$\alpha^{ap}$	Probability an infection is symptomatic given ACV	1/e	
$\alpha^{wp}$	Probability an infection is symptomatic given WCV	1	
ω	Waning from natural immunity	30 years	[16]
$\omega_{ap}$	Loss of immunity ACV	18 years	[16]
$\omega_{wp}$	Loss of immunity WCV	30 years	[16]
$c_{j}$	Population coverage	95%	[21]
p	Case report rate	1.5%	[29,33]
$\epsilon_{ap}$	ACV efficacy	$\epsilon_{1}=0.55,\;\epsilon_{2}=0.75,$ $\epsilon_{3}=0.84,\;\epsilon_{>4}=0.98$	[29]
$\epsilon_{wp}$	WCV efficacy	99%	[29]
$\gamma_{s}$	Symptomatic infection healing rate	25 days	[29]
$\gamma_{a}$	Asymptomatic infection healing rate	8 days	[29]

**Table 2:** Estimated parameters. In each realization of our simulation, the four parameters were drawn from the joint probability distribution, which was evaluated using the Monte Carlo Markov Chain algorithm.

Parameter	Prior Distribution	Posterior (Median Value, 95% HDI]
ϕ	U(0,2π)	2.93[1.513,3.953]
$f_{1}$	U(0,0.5)	$8.67\left[5.69,10.86\right]\cdot 10^{-4}$
$f_{2}$	U(0,0.5)	$2.67\left[2.22,4.79\right]\cdot 10^{-4}$
$f_{3}$	U(0,0.5)	$6.63\left[4.19,8.54\right]\cdot 10^{-5}$

**Table 3:** Cost-effectiveness parameter values. All costs and QALYs were discounted with a rate of 3.5%.

Parameter	Cost Per Episode (USD)	QALY Lost Per Episode	Justification
Vaccine	21		[39,40]
Home treatment	0	0.0022	[29]
Outpatient Visit	61	0.0068	[29,35,40]
Hospitalization	605	0.0115	[29,35,40]
Death	605	80	[29,35,40]

## Data Availability

All data used in the study are publicly available.
